# Undernutrition and its predictors among tuberculosis patients in Southwest Ethiopia

**DOI:** 10.3389/fnut.2024.1450669

**Published:** 2024-12-09

**Authors:** Nigusie Shifera, Tewodros Yosef

**Affiliations:** School of Public Health, College of Medicine and Health Sciences, Mizan-Tepi University, Mizan Teferi, Ethiopia

**Keywords:** tuberculosis, undernutrition, southwest region, Ethiopia, public hospitals

## Abstract

**Background:**

Adult tuberculosis (TB) patients experience significant undernutrition globally, especially in developing countries. While some studies have explored the prevalence and factors influencing undernutrition in this group, comprehensive large-scale investigations covering diverse health facilities and populations are lacking. This study aims to evaluate the prevalence of undernutrition and its associated factors among adult TB patients in public hospitals in southwest Ethiopia.

**Method:**

An institution-based cross-sectional study design was conducted from March 01 to April 15, 2023, in public hospitals in the southwest region, of Ethiopia. A total of 239 adult TB patients who were directly observed in TB treatment were selected via systematic sampling. A structured questionnaire was adapted from a review of different literature. Data were cleaned and entered into EPI info version 7, then analyzed with SPSS Version 22. A bivariable analysis was done to evaluate associations at (*p* < 0.25), and then multiple logistic regression models were computed to identify independent predictors of undernutrition among TB patients at (*p* < 0.05).

**Results:**

A total of 239 respondents participated with a response rate of 100%. The prevalence of undernutrition among adult TB patients was 43.93%. Of the 239 TB patients, the majority (91.6%) were new TB cases. Family size >5 (AOR 3.23 [1.16–9.01]), household average income <2,000 birr (AOR 5.64 [2.12–14.99]), type of TB (AOR 2.8 [1.25–6.51]), and positive HIV status of the study participant (AOR 3.23 [1.16–9.01]) were the independent predictors of undernutrition among adult TB patients.

**Conclusion and recommendations:**

Undernutrition among adult tuberculosis (TB) patients is notably high compared to other settings. Key predictors include a family size greater than five, a monthly income below 2,000 birr, HIV status, and pulmonary TB. Early screening and diagnosis of undernutrition, along with nutritional interventions, should be integrated into the routine care for all adult TB patients.

## Introduction

Tuberculosis (TB) is an infectious disease caused by *Mycobacterium tuberculosis* species and is transmitted mainly via coughing (1). Worldwide, TB is one of the top 10 causes of death and the leading cause of a single infectious agent, surpassing HIV/AIDS. It affects 10 million people and claims the lives of 1.3 million individuals every year. However, TB is curable and preventable. Approximately 85% of people who develop TB disease can be successfully treated with a 6-month drug regimen, and regimens ranging from 1 to 6 months can be used to treat TB infection (1, 2).

TB can affect anyone, regardless of age or gender. Adult men bear the highest burden, representing 56% of all TB cases in 2020. Adult women accounted for 33% of cases, while children made up 11%. The majority of TB patients, over 80%, reside in low-and middle-income countries, particularly in Asia and sub-Saharan Africa (2). In Ethiopia, according to the World Health Organization, it ranks seventh among the 22 High-burden countries globally. Furthermore, TB is one of the leading causes of hospital admission and death among adults in Ethiopia (3).

Undernutrition and active tuberculosis have a reciprocal relationship. Undernutrition significantly weakens the immune system, making individuals more susceptible to *Mycobacterium tuberculosis* infection and exacerbating disease progression (4). Nutrient deficiencies can impair the body’s ability to mount an effective immune response, leading to increased bacterial replication and severity of tuberculosis (TB). Additionally, the inflammatory response associated with TB can further deplete essential nutrients, creating a vicious cycle that hinders recovery and treatment outcomes (5).

Studies have consistently found that undernutrition is associated with increased tuberculosis incidence, increased severity, worse treatment outcomes, and increased mortality (6). The risk of acquiring TB increases by 13.8% for each unit decrease in body mass index (BMI), although this relationship is not maintained at the extremes of the BMI range (7). Approximately 25% of new tuberculosis (TB) cases worldwide are attributed to undernutrition (5). undernourished patients are twice as likely to die from TB compared with non-malnourished patients (8). Moreover, Patients with undernutrition had a two times higher risk of experiencing unsuccessful treatment outcomes compared to well-nourished patients (9).

Improving the nutritional status of TB patients is a critical step towards the reduction of mortality due to TB. Studies have confirmed that optimized nutritional treatment has shown beneficial effects on patient prognosis, and the provision of nutritional supplementation alongside treatment is associated with increased treatment compliance. WHO also recommends nutritional treatment, including counseling, for all patients with TB following an assessment for malnutrition at diagnosis and routinely (every 4 weeks) during treatment (10).

In Ethiopia, few studies have assessed the extent of undernutrition among TB patients, showing prevalence rates between 28.5 and 71.35% (11, 12). A recent systematic review found that 48.23% of TB patients are undernourished (13). However, no large-scale investigation has been conducted across multiple health facilities that encompass diverse populations and socio-cultural backgrounds. Moreover, the majority of previous studies did not adequately address behavioral factors, food access, the duration of the disease before diagnosis, the history of prior therapy, and hygiene factors. Furthermore, this study has not been conducted in southwest Ethiopia, even though undernutrition is a persistent nutritional problem in the community. This study aimed to assess undernutrition and its associated factors among adult tuberculosis (TB) patients in public hospitals in southwest Ethiopia. Understanding the extent of undernutrition in this population is crucial for developing targeted interventions to address their nutritional needs (14).

## Method and materials

### Study area, period, and design

An institution-based cross-sectional study design was conducted from March 01 to April 15, 2023, at public health facilities in the South West Ethiopia Peoples’ Regional State. The region has six administrative zones and four regional capital cities namely Bonga (449 km from Addis Ababa), Mizan-Aman (561 km from Addis Ababa), Tepi, and Tarcha. The Region has an estimated population of 3,374,706, of which 1,670,479 are male and 1,704,227 are female, with 688,716 households.

There are seven hospitals in the region: one referral teaching hospital (MTUTH), two general hospitals (Gebretsadik Shawu and Tepi General Hospitals), and four primary hospitals. In 2014 EFY, the estimated number of registered TB cases was 1,241. These hospitals provide health services and serve as referral centers for district primary hospitals and health centers catering to over five million people.

The region’s geography features a humid tropical climate with a Woina Dega (highland fringe) classification, characterized by temperatures between 20 and 25 degrees Celsius, rainfall of 1,200–2,800 mm, and an altitude range of 880 to 3,360 meters. The region has 27% natural forest coverage. The South West Peoples’ Region boasts significant agricultural potential for a variety of crops, including coffee, as well as extensive biodiversity due to its forest coverage.

### Population

All adult TB patients aged 18 years and above who are on DOTS (Directly Observed Treatment, Short-course) in TB clinics of public hospitals in the Southwest region comprised the source population. Additionally, all adult TB patients on DOTS (Directly Observed Treatment) in TB clinics during the data collection period who fulfilled the eligibility criteria were considered the study population.

### Eligibility criteria

All TB patients who were on DOTS (Directly Observed Treatment) and aged 18 years and above were included in this study. Patients with any anatomical deformities, as well as pregnant and lactating women, were not included in this study. Moreover, TB patients who were critically ill and unable to communicate were also excluded from the study.

### Sample size determination

For the first objective, the sample size was determined considering the following assumptions using the single population formula: a Z-score at a 95% confidence interval of 1.96, a margin of error of 5%, and a prevalence of undernutrition among TB patients in Hossana estimated at 39% (40 patients), resulting in a sample size of 366. Then, a 5% non-response rate was added, increasing the sample size to 386. As the number of TB patients who had follow-ups at public health facilities in the study area was less than 10,000, a correction formula was used, yielding a final sample size of 231.

For the second objective, the sample size was calculated by the double population proportion formula using Epi info 7.2. A study conducted in Addis Ababa found nutritional counseling services, and TB/HIV co-infection to be factors associated with undernutrition among TB patients. By considering these factors, 95% confidence interval, 80% power, 1:1 ratio of exposed to the unexposed percentage of outcome in unexposed, and 5% non-response rate, from the above calculation the larger sample size was 239. Then, after comparing the sample sizes from both objectives, the largest sample size, which is 239, was taken as the final sample size for this study.

### Sampling procedures

In the Southwest region of Ethiopia, seven hospitals were identified, and three were randomly selected for this study: Mizan Tepi University Teaching Hospital, Tepi General Hospital, and Gebretsadik Shawu General Hospital. The total number of adult tuberculosis (TB) patients in these hospitals was 621, with 240 patients from Mizan Tepi Hospital, 165 from Tepi Hospital, and 261 from Bonga Hospital.

To ensure representation, the sample size was proportionally allocated to each hospital based on the number of TB patients. As a result, 131 participants were chosen from Mizan Tepi University Teaching Hospital, 90 from Tepi General Hospital, and 118 from Bonga Hospital. This selection was carried out using a systematic sampling technique, with a k value calculated as 621 divided by 239, resulting in a *k* value of 2. The first participant was selected using a simple random sampling technique.

### Data collection technique

A structured questionnaire was adapted from a review of different literature (6, 9, 11, 14). Data collectors were nurses who work in the TB unit, and training was given for 1 day, particularly in the proper filling of the questionnaire and also emphasizing anthropometric measurements (i.e., instrument calibration & the use of weight and height scale), to minimize inter and intra-observer errors. The questionnaire was prepared in English and then translated into Amharic. It was back-translated to English by professionals to check for consistency.

Standard techniques of anthropometric measurements were used. Anthropometric measurements, particularly weight and height, were taken from all study participants by nurses under the close supervision of the Principal Investigator. Weight was measured using a portable standing scale and recorded to the nearest 0.1 kg. During the procedure, participants wore light clothing and were barefoot. Height in centimeters was marked on a wall with the help of a measuring tape. All subjects were measured against the wall without footwear, with heels together and heads positioned so that their eyes were looking straight ahead, ensuring the line of vision was perpendicular to the body. The wooden scale was brought down to the highest point on the head, and height was recorded to the nearest 0.1 cm. The same measurer was employed for each anthropometric measurement to avoid variability. Body mass index (BMI) was calculated by dividing weight (in kg) by height (in m^2^) (15).

### Variables

#### Dependent variable

Nutritional status: undernutrition (Yes: Body mass index <18.5 kg/m^2^; No: Undernourished BMI ≥ 18.5 kg/m^2^).

#### Independent variables

Socio-demographic characteristics: Age, Sex, Family size, Residence, Educational status, Income of the household.

Nutritional factors: Provision of Food supplements, Nutritional counseling, Nutrition care and support, Poor eating problem.

TB–related factors: Type of TB- (PTB, Extra PTB), History of previous treatment, Duration of TB treatment.

Clinical factors: TB-HIV co-infection, History of other chronic disease (DM, HPT….)

Behavioral and lifestyle-related factors: Physical exercise, Alcohol intake, Cigarette smoking, Khat use.

### Operational definition

Undernutrition: Undernutrition is defined as Body mass index<18.5 kg/m2 and not undernourished BMI ≥18.5. Kg/m2 (16).

Nutritional care: Has components such as receiving nutritional education to both patient and caregiver, performing nutritional screening, receiving encouragement to eat healthy food and maintain a healthy weight, manage if nutritional problems occur. If the participant gets at least one of the care we consider it as received nutritional care (17).

Nutritional support: Have a component optimize patient oral intake, provide nutritional supplements, and administer enteral and parental nutrition. If the participant gets at least one of the support we consider it as received nutritional support (18).

Dietary counseling: Two-way interaction through which a client and a trained counselor interpret the results of dietary assessment, identify individual nutrition needs and goals, discuss ways to meet those goals, and agree on the next steps (19).

Poor eating problems: Were taken as if the patient reported one of the following during data collection mouth ulcer, poor apatite, nausea or vomiting, and difficulty of swallowing.

### Data processing and analysis

Data were checked for completeness and consistency, and then edited, coded, and entered using Epi info version 7 then exported to SPSS version 27 and checked for missing values before analysis. Descriptive statistics were computed, and tables and figures were used to determine the frequencies of the dependent and independent variables. The prevalence of undernutrition was determined by the ratio of the number of patients with undernutrition to the total sample size.

A bivariable analysis was done to evaluate the associations of individual explanatory variables with the dependent variables. Variables that showed an association with the outcome variables in the bivariate analyses (*p* < 0.25) were entered into multiple logistic regression models to identify independent variables that have a significant association with undernutrition among TB patients. Independent predictors in the multivariable logistic regression were declared significant at (*p* < 0.05) with a 95 percent confidence level. The Hosmer-Lemeshow goodness-of-fit statistic was used to check if the necessary assumptions for multiple logistic regressions were fulfilled and the model had a *p*-value >0.05 which proved the model was good.

### Data quality management

Data collectors were nurses who work in the TB unit and training was given for 1 day, particularly in the proper filling of the questionnaire, and the use of the weight and height scales to minimize inter and intra-observer errors. The questionnaire was prepared in English and then translated into Amharic and back-translated to English by professionals to check for its consistency. Before the actual data collection, a pretest was done on 5% of the sample size. During data collection time, close monitoring was done by the investigators. Data from each respondent were checked for completeness. In addition, the data were thoroughly cleaned and carefully entered into the computer by the Epi data manager using double-entry verification.

### Ethical consideration

Ethical approval and clearance were obtained from Mizan-Tepi University. Letters of cooperation were taken from each hospital. Written consent was obtained from TB patients after a clear explanation was given about the aim of the study. Confidentiality and privacy were maintained during data collection by interviewing participants in a separate room, and during analysis and reporting, where the information obtained from the respondents was not shared with anyone other than the data collectors and principal investigator. Respondents with a problem of undernutrition were counseled and referred to the adult outpatient department (OPD) for appropriate nutritional care and support.

## Results

### Socio-demographic characteristics

A total of 239 respondents participated in the study, achieving a 100% response rate. The majority of the participants were male, accounting for 152 individuals (63.6%), while the mean age of the participants was 33.6 (±13.26). In terms of educational status, 47 (19.64%) had no education, 90 (37.6%) attended primary school, and only 6.3% had education beyond secondary school. Nearly half of the participants, 106 (44.35%), had an average monthly income between 2000 and 4,000 Ethiopian Birr (Table 1).

**Table 1:** Socio-demographic characteristics of adult TB patients in public hospitals of the Southwest Region, Ethiopia, 2023 (*N* = 239).

Variables	Categories	Frequency (*N*)	Percent (%)
Age	18–24 years	68	28.45
	25–34 years	77	32.22
	35-44 years	43	17.99
	>45–60 years	51	21.34
Sex	Male	152	63.60
	Female	87	36.40
Marital status	Married	164	68.62
	Single	66	27.62
	Widowed/separated	9	3.76
	No formal education	47	19.6
	Can write and read	48	20.1
	Primary education	90	37.7
	Secondary education	39	16.3
	Above secondary	15	6.3
Family size	Less than 5	152	63.6
	Greater than or equal5	87	36.4
Religion	Orthodox	75	31.38
	Protestant	129	53.97
	Muslim	35	14.65
Residency	Urban	146	61.09
	Rural	93	38.91
Occupation	Farmer	61	25.52
	Merchant	50	20.92
	Daily laborer	44	18.41
	Govt employ	23	9.62
	Housewife	35	14.64
	Other	26	10.88
Average monthly income (ETB or birr)	<2000	72	30.13
	2000–4,000	106	44.35
	>4,000	61	25.52

### Nutritional and diet characteristics

About 101 participants (42.31%) used their food products, while 73 (30.5%) relied on a combination of their own and market-purchased products. The majority of participants, 216 (90%), had three meals per day. Only 95 participants (40%) received nutritional care, with 59 (62.1%) receiving encouragement regarding their food choices and 35 (36.8%) receiving nutritional counseling. Additionally, less than 13% received nutritional support (Table 2).

**Table 2:** Nutrition and diet information of adult TB patients in public hospitals of the Southwest Region, Ethiopia, 2023 (*N* = 239).

Variable	Categories	Frequency	Percent
Source of food for consumption.	Own product only	101	42.31
	Market purchase only	65	27.19
	product and market purchase	73	30.5
Number of meals per day	Two meal only or bellow	9	3.77
	Three meal	216	90.38
	Above three meal	14	5.86
Nutrition care during treatment	Yes	95	39.75
	No	144	60.25
Type of nutritional care	Nutritional education	35	36.84
	Nutritional screening	1	1.05
	Encouragement to eat	59	62.11
Nutritional support	Yes	30	12.55
	No	209	87.45
Type of nutritional support	Optimize patient oral intake	23	76.67
	Nutritional supplementation	6	20
	Administer enteral and parental	1	3.33
Get regular dietary|Counseling	Yes	224	93.72
	No	15	6.28

### Behavioral and lifestyle characteristics

Out of the participants, 210 (87.87%) had no history of physical exercise, while 161 (67.36%) reported no history of alcohol intake. Furthermore, 218 (91.2%) had no history of smoking, but 66 (27.6%) had a history of alcohol consumption, and 26 (10.8%) reported chewing khat (Table 3).

**Table 3:** Behavior and lifestyle status of adult TB patients in public hospitals of the Southwest Region, Ethiopia, 2023 (*N* = 239).

Variables	Categories	Frequency	Percent
History of physical exercise per day	30 min	18	7.53
	Less than 30	4	1.67
	More than 30	7	2.93
	None	210	87.87
Smoking status	Current smoker	1	0.42
	Former smoker	20	8.37
	Never smoke	218	91.21
Alcohol intake	Yes	78	32.64
	No	161	67.36
Frequency of alcohol intake in the past 30 days	Once	14	17.95
	Two	14	17.95
	Three and above	50	64.10
History of khat chewing	Yes	26	10.88
	No	213	89.12
Frequency of khat chewing	Every day	5	19.23
	Once per week	21	80.77

### Clinical characteristics

Out of the total study participants, 6% reported having another TB patient in their household. Nearly half of the participants, 125 (52.30%), experienced eating problems, and among them, 98.4% had poor appetite. Out of the 239 study participants, 63 (26.36%) reported experiencing an illness other than TB. Among these participants, 36 (57.1%) had a fever, and 9 (14.29%) had diarrhea (Table 4).

**Table 4:** General health status of adult TB patients in public hospitals of the Southwest Region, Ethiopia, 2023 (*N* = 239).

Variables	Categories	Frequency	Percent
TB patient in the house	Yes	15	6.28
	No	224	93.72
Problem with eating	Yes	125	52.30
	No	114	47.70
Type of eating problem	Poor appetite	123	98.40
	Nausea and vomiting	2	1.60
Current experience any illness	Yes	63	26.36
	No	176	73.64
Type of illness	Fever	36	57.14
	Diarrhea	10	15.88
	HPN	17	26.98

### Nutritional status

**Figure 1:**
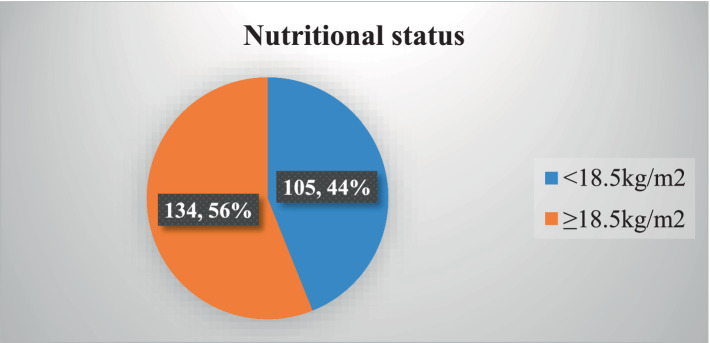
Nutritional status among adult TB patients in public hospitals of the Southwest Region, Ethiopia, 2023 (*N* = 239).

The prevalence of undernutrition (BMI of <18.5 kg/m^2^) among the participants was 43.93% (Figure 1).

### TB status-related characteristics

Among the 239 study participants, 99 (41.42%) were diagnosed with smear-positive pulmonary TB, while 69 (28.8%) had extrapulmonary TB. Most of the participants were new TB cases (Table 5).

**Table 5:** TB status of patients from health institution records of adult TB patients in public hospitals of the Southwest Region, Ethiopia, 2023 (*N* = 239).

Variable	Categories	Frequency	Percent
TB status	New TB cases	219	91.6
	Relapse	19	7.9
	Treatment failure	1	0.5
Type of Tb	Smear positive PTB	99	41.42
	Smear negative PTB	71	29.71
	Extra PTB	69	28.87
HIV status	Positive	29	12.13
	Negative	197	82.43
	Not record	13	5.44
Duration on treatment	<2 weeks	42	17.57
	>2 weeks	197	82.43

### Multivariable analysis

In the multivariable analysis, four variables were found to have a significant association with the nutritional status of the study participants: family size, average income, and the type of TB and HIV status. TB patients living in households with a family size of ≥5 people had 3.19 times higher odds of experiencing undernutrition compared to those with smaller family sizes (AOR = 3.19, 95% CI: 1.603–9.01). TB patients with an average income of less than 2,000 birr had 5.64 times higher odds of developing undernutrition compared to those with an average income greater than 4,000 birr (AOR = 5.64, 95% CI: 2.12–14.99).

Being a smear-positive TB patient was associated with 2.8 times higher odds of developing undernutrition compared to having extrapulmonary TB (AOR = 2.8, 95% CI: 1.25–6.51). Similarly, being HIV-positive was associated with 3.23 times higher odds of developing undernutrition compared to being HIV-negative and having TB (AOR = 3.23, 95% CI: 1.16–9.01) (Table 6).

**Table 6:** Multivariable logistic regression variables to identify independent predictors of undernutrition among TB patients in public hospitals of the Southwest Region, Ethiopia, 2023 (*N* = 239).

Variables	Nutritional status (BMI)		COR (95%CI)	AOR (95%CI)	*p*-value
	BMI < 18.5, *n* (%)	BMI ≥ 18.5, *n* (%)			
Occupational status					
Farmer	34 (32.38)	27 (20.15)	8.4 (2.25–31.25)	1.7 (0.219–13.88)	0.598
Merchants	26 (24.76)	24(17.9)	7.2 (1.9–27.42)	3.7 (0.53–26.38)	0.184
Daily laborer	19 (18.1)	25 (18.6)	5.06 (1.31–19.58)	1.7 (0.22–14.07)	0.589
Housewife	12 (11.4)	23 (17.1)	3.47 (0.858–14.1)	0.83 (0.10–6.79)	0.870
Other	11 (10.4)	15 (11.1)	4.88 (1.15–20.66)	2.1 (0.29–15.42)	0.463
Gov. employee	3 (2.8)	20 (14.9)	1	1	
Educational status					
Not read and write	31 (29.5)	16 (11.9)	12.59 (2.53–62.77)	3.2 (0.84–12.38)	0.087
Read and write	22 (20.9)	26 (19.4)	5.7 (1.11–27.05)	1.23 (0.36–4.15)	0.738
Primary	38 (36.1)	52 (38.8)	4.75 (1.01–22.29)	1.34 (0.45–3.96)	0.589
Secondary	12 (11.4)	27(20.1)	2.8 (0.56–14.84)	1.52 (0.15–16.10)	0.729
Above Secondary	2 (1.9)	13(9.7)	1	1	
Family size					
≥ 5 people	55 (52.38)	32 (23.88)	2.57 (1.49–4.41)	3.19 (1.16–9.01)*	0.001
<5 people	50 (47.62)	102 (76.12)	1	1	
Average income					
<2000 birr	49 (46.6)	23 (17.4)	8.3 (3.3–16.18)	5.64 (2.12–14.99)*	0.001
2000–4,000 birr	44 (41.9)	62(46.27)	2.7 (1.2–5.4)	3.01 (1.22–7.49)*	0.017
>40,000 birr	12 (11.4)	49 (36.57)	1	1	
Number of meals per day					
Two times	7 (6.6)	2 (1.4)	12.8 (1.69–97.19)	2.11 (0.35–12.714)	0.414
Three times	95 (90.4)	121 (90.3)	2.8 (0.78–10.61)	1.2 (0.65–2.45)	0.849
Four and above	3 (2.8)	11 (8.2)	1	1	
Dietary counseling					
No	11 (10.4)	4(2.9)	0.3 (1.17–12.31)	3.1 (0.65–14.83)	0.154
Yes	94 (89.5)	130 (97.01)	1	1	
Alcohol intake					
Yes	45 (42.8)	33 (24.63)	2.1 (0.64–1.9)	1.2 (0.646–2.41)	0.499
No	60 (57.14)	101 (75.3)	1	1	
Type of TB					
Smear positive	59 (56.2)	40 (29.8)	4.17 (2.13–8.17)	2.8 (1.25–6.51)*	0.012
Smear negative	28 (26.6)	43 (32.1)	1.8 (0.89–3.78)	1.15 (0.49–2.703)	0.733
Extra pulmonary	18 (17.1)	51 (38.1)	1	1	
HIV status					
Positive	21 (20)	8 (5.9)	3.9 (1.65–9.290)	3.23 (1.16–9.01)*	0.025
Not recorded	5 (4.7)	8 (5.9)	0.93 (0.29–2.9)	0.67 (0 0.17–2.61)	0.573
Negative	79 (75.2)	118 (88.06)	1	1	

*indicates a variable with *p*-value < 0.05 in the multivariate analysis.

## Discussion

The prevalence of undernutrition among adult TB patients in our study was 43.93%, which is similar to the findings from Jijjega, East Ethiopia (44.3%) (20), Ghana (51%) (21), and Adama (53%) (22). However, the above-stated prevalence is higher than the rates reported in a study conducted in Hosahana, South Ethiopia (38.9%) (23), and Addis Ababa (39.7%) (11). Although Ethiopia has made significant progress in reducing undernutrition among TB patients, further attention is still needed. Early screening and diagnosis of tuberculosis, along with assessing patients’ nutritional status, should be integral to the routine care of all adult TB patients. On the other hand, our study’s prevalence is lower compared to another study conducted in the Amhara region of Ethiopia (57.17%) (24) and the Bale zone of Ethiopia (63.2%) (25). The difference in our study’s results compared to others could be because we focused specifically on TB patients who were receiving anti-TB medication and follow-up care, whereas other studies may have included all TB patients regardless of their treatment status. Moreover, variations in the methods used to diagnose undernutrition could also contribute to the difference in prevalence rates.

TB patients of family size ≥5 were 3.2 times higher risk of of experiencing undernutrition compared to those with smaller family sizes. A study conducted in Metema and Hosanna, South Ethiopia, found that leaving a large and extended family was associated with undernutrition in adult TB patients (23, 26). Furthermore, a Gahana study found a negative correlation between family size and undernutrition (27). This may be due to; high family size decreasing the household income and leading to the low dietary intake of household members (28). Policy needs to focus on household wealth and fertility rates. Additionally, nutritional support for patients is also required.

The odds of undernutrition were about 5.6 times more likely among TB patients who had average monthly income (< 2000 ETB) compared to income greater than (>4,000 ETB). A study conducted in Ghana, Brazil, and Ethiopia, specifically in Adama and Tigray, showed that the likelihood of participants being undernourished increased when their average monthly income was low (21, 22, 29). The observed association suggests that people with low income could not afford food, which might contribute to food insecurity, reduced intake, and nutrient deficiencies (30). Moreover, TB disease by itself is a disease that mainly affects poor people because of their low standard of living (10).

TB and HIV infections are both independently associated with undernutrition. However, co-infection with HIV can worsen the extent of undernutrition, For instance, a study conducted in Gondar revealed that the prevalence of undernutrition among adult TB patients co-infected with HIV was 71.6%, which is higher compared to the prevalence of undernutrition among TB patients without HIV (31). This study also found that TB patients with HIV infection had 3.2 times higher odds of developing undernutrition compared to those without HIV infection. These findings are consistent with studies conducted in Adama Town and Hosahana in the SNNPR region of Ethiopia, which showed a significant association between HIV co-infection and undernutrition (22, 23). This could be attributed to the fact that TB/HIV co-infected patients are more vulnerable to undernutrition due to the double burden they face (32). Furthermore, HIV co-infection leads to poor economic productivity (33), reduced appetite, and impaired absorption, all of which contribute significantly to undernutrition (34).

The odds of undernutrition among pulmonary-positive adult TB patients were 2.8 times those of extrapulmonary TB cases. This is consistent with a study conducted in North Ethiopia (29) and low BMI was found among pulmonary-positive patients in Kenya (35). However, our result is not consistent with studies done in Shashemane (36), Addis Ababa (11), Ethiopia and Nepal (37). This could be due to the diagnosis of extra-pulmonary tuberculosis (TB) is more challenging compared to pulmonary TB, and patients are often not detected and treated early enough.

## Conclusion and recommendation

This research has found that malnutrition is prevalent among adult tuberculosis (TB) patients in southwest Ethiopia. Moreover, family size, average monthly household income, co-infection with TB, and extrapulmonary TB were identified as contributors to undernutrition among TB patients.

Regular assessment of nutrition, providing dietary counseling, education, and nutritional intervention should be part of the routine care for all adult TB patients. Special attention must be directed towards patients co-infected with TB and HIV, as well as those with extrapulmonary disease. Nutritional counseling should be tailored to help increase their energy intake. Providing nutritional supplements alongside counseling is essential for their recovery. This targeted approach can significantly enhance their overall health and treatment outcomes.

To support low-income families facing undernutrition in TB patients, implement community-based meal programs that offer nutritious, affordable food options. Facilitate access to local food banks and nutrition assistance programs tailored to these families.

Policymakers, along with both governmental and non-governmental organizations, should take the aforementioned factors into account when developing and delivering nutritional services for TB patients. This ensures that the services are effective and adequately meet the needs of those affected. Collaboration and careful planning are essential for optimal outcomes. Ultimately, addressing these considerations will improve patient care and recovery rates.

### Limitation of the study

Besides addressing an important area of research that could provide input for preventing health problems related to undernutrition in adult TB patients, the study did not use other biomarkers and methods to classify undernutrition. Moreover, the questions used to assess behavioral and lifestyle factors may have been subject to social desirability bias.

The study is not without any limitations. The study used only the BMI to classify undernutrition. Additionally, variables like Food biodiversity were not assessed. We recommend other researchers to consider this issue while using our findings.

## Data Availability

The raw data supporting the conclusions of this article will be made available by the authors, upon reasonable request.
